# Spatio-Temporal Dynamics of Maize Potential Yield and Yield Gaps in Northeast China from 1990 to 2015

**DOI:** 10.3390/ijerph16071211

**Published:** 2019-04-04

**Authors:** Luoman Pu, Shuwen Zhang, Jiuchun Yang, Liping Chang, Shuting Bai

**Affiliations:** 1 College of Earth Sciences, Jilin University, Changchun 130012, China; puluoman@sina.com (L.P.); baist2018@163.com (S.B.); 2 Northeast Institute of Geography and Agroecology, Chinese Academy of Sciences, Changchun 130102, China; lpchang@iga.ac.cn; 3 Department of Microbiology and Plant Biology, Center for Spatial Analysis, University of Oklahoma, Norman, OK 73019, USA

**Keywords:** spatio-temporal dynamics, maize potential yield, Northeast China, yield gap, GAEZ model

## Abstract

Maize yield has undergone obvious spatial and temporal changes in recent decades in Northeast China. Understanding how maize potential yield has changed over the past few decades and how large the gaps between potential and actual maize yields are is essential for increasing maize yield to meet increased food demand in Northeast China. In this study, the spatial and temporal dynamics of maize potential yield in Northeast China from 1990 to 2015 were simulated using the Global Agro-ecological Zones (GAEZ) model at the pixel level firstly. Then, the yield gaps between actual and potential yields were analyzed at city scale. The results were the following. (1) The maize potential yield decreased by about 500 kg/ha and the potential production remained at around 260 million tonnes during 1990–2000. From 2000 to 2015, the maize potential yield and production increased by approximately 1000 kg/ha and 80 million tonnes, respectively. (2) The maize potential yield decreased in most regions of Northeast China in the first decade, such as the center area (CA), south area (SA), southwest area (SWA), and small regions in northeast area (NEA), due to lower temperature and insufficient rainfall. The maize potential yield increased elsewhere. (3) The maize potential yield increased by more than 1000 kg/ha in the center area (CA) in the latter 15 years, which may be because of the climate warming and sufficient precipitation. The maize potential yield decreased elsewhere and Harbin in the center area (CA). (4) In 40 cities of Northeast China, the rates of actual yield to potential yield in 17 cities were higher than 80%. The actual yields only attained 50–80% of the potential yields in 20 cities. The gaps between actual and potential yields in Hegang and Dandong were very large, which need to be shrunk urgently. The results highlight the importance of coping with climate change actively, arranging crop structure reasonably, improving farmland use efficiency and ensuring food security in Northeast China.

## 1. Introduction

At present, the world is experiencing rising demands for crop production, stemming from three key forces: increasing human population, meat and dairy consumption from growing affluence, and biofuel consumption [1,2]. The only peer-reviewed estimate suggests that crop demand may increase by 100%–110% between 2005 and 2050 [2]. Therefore, in order to improve crop production to satisfy the growing population and food and biofuel needs, it is crucial to understand the magnitudes and causes of potential yield and yield gaps between potential yield and actual yield achieved by farmers. Improving the yield (per unit area) of existing agricultural land, not expanding the area of cropland at the expense of other ecosystems, is a high priority.

In recent decades, the crop production and spatio-temporal distribution have changed greatly, and the spatio-temporal dynamics of crop potential yield and yield gaps have become major research topics. Determination of the potential yield and the gaps between potential and actual yields requires a thorough understanding of crop growth and development, which in turn depends on climatic, edaphic, hydrological, physiological, and management factors [3]. Much previous research have been done on the spatial and temporal changes of crop potential yield and yield gaps. Qin et al. investigated climate change during 1961–2010 and the spatial and temporal characteristics of climate resources in newly converted cropland during 1990–2010 across northern China and drew the conclusion that the average climate potential productivity of newly converted cropland decreased considerably from 672.41 to 440.40 t/km^2^, indicating a substantial decline in the quality of newly converted cropland [4]. Zhang et al. analyzed the spatial and temporal changes in the frequency of major agrometeorological disasters affecting maize production and found the frequency of disasters affecting maize increased significantly during the reproductive growth period than the vegetative growth period [5]. Ji et al. analyzed the facts of climate change and its effects on the maize production in Northeast China according to the meteorological, maize yield, and planting area data and found that with the heat resources increasing continually, adaptive area of maize planting is growing, with its north boundary extending northward and eastward, so the adaptive seeding date comes earlier [6]. Tao et al. estimated maize yield potentials from 1980 to 2008 across the major maize production regions of China by county and analyzed the yield gaps [7]. Licker et al. compared the actual yield to climate-specific attainable yield and showed that maize in eastern China generally had large yield gaps [8]. Mueller et al. applied a global-scale assessment of intensification prospects method and drew a conclusion that large production increases (45% to 70% for most crops) are possible from closing yield gaps to 100% of attainable yields [9]. Although the above studies have tried to quantify the potential yield, it is still difficult to obtain reliable field-based quantifications of potential yield due to the lack of observations associated with perfect crop management. When such data are lacking, crop modeling is considered as the most effective means to estimate crop potential yield because it allows the assessment of the interactive impacts of climate, crop cultivar, and crop management on crop growth and development [10,11,12]. Therefore, the highlight of this study is to quantify the crop potential yield by using a simulation model and explore the yield gaps by comparing potential yield and actual statistical data. Quantifying the yield gaps is essential to identify the possible degree of yield improvement attainable in the near future to ensure food security in China.

Maize is one of the common crops in Northeast China. As the second largest maize producer and consumer, China accounts for more than 20% of total production annually in the world [3]. The production of maize in Northeast China accounts for a third of China’s maize production [13]. Therefore, the objectives of this study are: (1) to use the process-based model, Global Agro-ecological Zones (GAEZ) model to simulate maize potential yield for three years based on Digital Elevation Model (DEM) data, meteorological data, soil data, farmland, and irrigation data; (2) to study the spatial and temporal dynamics of maize potential yield in Northeast China during 1990–2015 and further analyzed the reasons; (3) to investigate the yield gaps between maize actual and potential yields at the city scale. The results of this research can provide scientific basis and guidelines for arranging the grain planting structure reasonably and formulating relevant management regulations in Northeast China.

## 2. Data and Methods

### 2.1. Study Area

**Figure 1:**
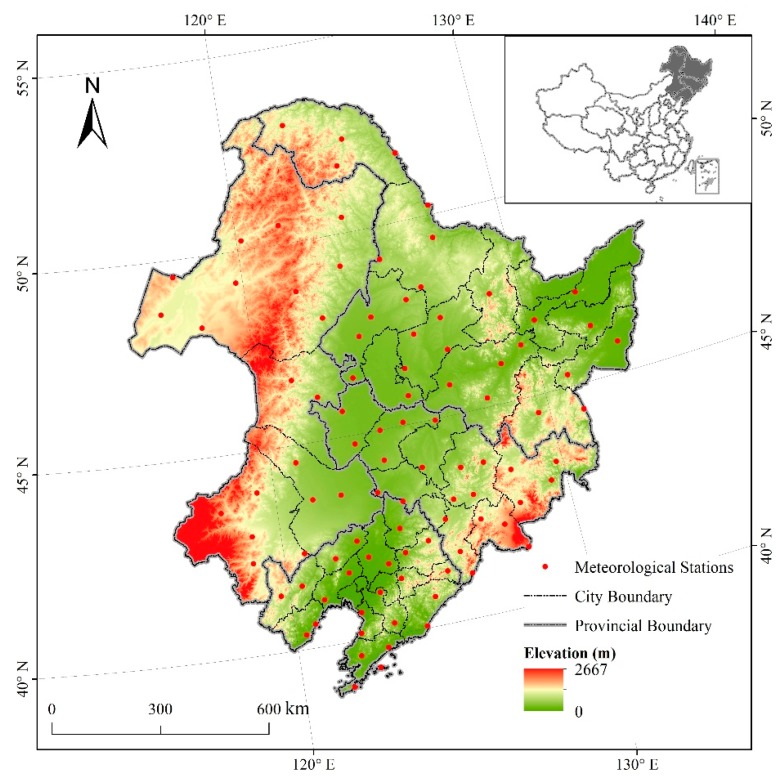
Location of the study area in China.

Northeast China extends from 38°40′ N to 53°34′ N, and 115°05′ E to 135°02′ E covering Heilongjiang, Jilin, and Liaoning, as well as the eastern parts of the Inner Mongolia Autonomous Region (IMAR) (Figure 1). It consists of 40 cities with the total land area of about 1.24 million km^2^. The study area is surrounded by middle and low mountains along three directions, including the Changbai Mountains in the southeast, the Greater Khingan Mountains in the northwest, and the Lesser Khingan Mountains in the northeast. Some plains are located in the central and southern parts and in the northeastern corner [14]. The climate is influenced by the East Asian monsoon, which has four distinct seasons, with a long winter and a short summer [15]. The annual temperature ranges from −5 to 10.6 °C and ≥10 °C annual accumulated temperature is 2200–3600 °C. The frost-free period is 140–170 d [16]. The average annual precipitation, which is concentrated from July to September and represents 70% of the annual total, ranges from 1000 mm in the east to 350 mm in the west [17]. The corresponding main soil types in Northeast China are brown coniferous forest soils in the cold temperate zone, dark brown forest soil in the warm temperate zone, and forest steppe chernozem and meadow steppe chernozem in the temperate zone [18]. Northeast China is mainly occupied by farmland and forest, which cover 73.64% of the total area. Maize is the major crop in Northeast China. By referring to the Statistical Yearbook of Northeast China, the actual maize production accounts for about 70% of the total grain production (including cereal, tuber and soybean) in Northeast China in 2015 (Table 1) [19,20,21,22].

### 2.2. Data Source

#### 2.2.1. Input Data for the GAEZ Model

The input data for the GAEZ model in this study included meteorological data, soil data, terrain data, farmland data, and irrigation data. These data were reprojected to the World Geodetic System 1984 (WGS-84) coordinate system.

The meteorological data for three years (1990, 2000, and 2015) were obtained from the National Meteorological Information Center. These observations were from 99 meteorological stations distributed throughout the study region at a wide range of elevations (Figure 1). The meteorological variables included monthly mean maximum and minimum temperature, cumulative precipitation, cumulative solar radiation, mean relative humidity, mean wind speed at 10 m height, and wet day frequency (the number of days on which the precipitation exceeds 0.2 mm). The above seven kinds of variables related to crop growth were interpolated to 10 km spatial resolution raster data by using ANUSPLIN software based on the DEM of Northeast China [23,24,25].

The soil data of Northeast China were extracted from the corresponding grid cell in the 1 km × 1 km Harmonized World Soil Database (HWSD) developed by the International Institute for Applied Systems Analysis (IIASA) and Food and Agriculture Organization (FAO), which included various soil attributes such as soil texture, organic carbon content, soil acidity, soil drainage ability, and so on [26]. The soil data also need to be processed to 10 km resolution grid.

The terrain data, high-resolution raster DEM, were from the Shuttle Radar Topography Mission (SRTM) C-band data [27]. The DEM data with 90 m spatial resolution was processed into slope and aspect data and resampled to 10 km spatial resolution grid.

The farmland data were extracted from the land use database developed by the Chinese Academy of Sciences (with a mapping scale of 100,000) in years 1990, 2000, and 2015. The land use database was obtained from manual visual interpretation at Landsat Thematic Mapper/Enhanced Thematic Mapper (TM/ETM) and Operational Land Imager (OLI) images. Through field verification, the interpretation precision was >94.3%, which could satisfy the accuracy requirement of 1:100,000 mapping. The land use data were divided into six major categories, including farmland, woodland, grassland, water bodies, built-up land, and unused land. The farmland data need to be processed to farmland ratio data with 1 km spatial resolution grid.

The irrigation data for three years (1990, 2000, and 2015) were the irrigation area data of each city from the Statistical Yearbook of Northeast China [19,20,21,22]. They also need to be processed to irrigation ratio data with 1 km spatial resolution grid.

#### 2.2.2. Other Data

Other data included the statistical data of actual maize yield and production of each city in Northeast China from the Statistical Yearbook [19,20,21,22].

### 2.3. Methods

#### 2.3.1. Procedures for Calculating Potential Yield

**Figure 2:**
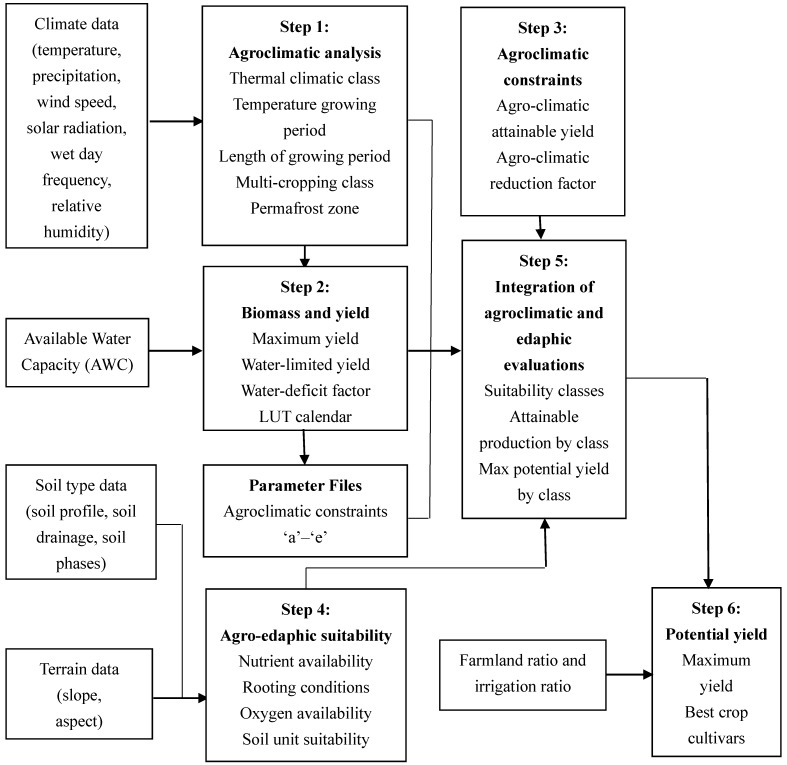
Procedures for calculating crop potential yield in the GAEZ model.

The procedures used in the GAEZ model to simulate maize potential yield are shown in Figure 2. The method of GAEZ is based on the AEZ approach developed by IIASA and FAO [28]. Since then, the Global AEZ model has been developed. In 2012, FAO in partnership with IIASA has released the latest version (3.0) of GAEZ database and data portal. The current GAEZ (GAEZ v3.0) provides a major update of data and extension of the methodology compared to the earlier version. It employs simple and robust crop models and provides standardized crop-modeling and environmental matching procedure to identify crop-specific limitations of prevailing climate, soil, and terrain resources under assumed levels of input and managements conditions [29]. The details of each procedure in the GAEZ model are as follows.

Step (1). Seven climatic variables are prepared to be interpolated to 10 km spatial resolution grid and then input to the model, which provide the basic for the calculation of soil water balances and agroclimatic indicators relevant to plant production. Agroclimatic indicators include wind speed at 2 m height [30], reference and actual evapotranspiration [31], snow balance calculation, etc. Then, the thermal regimes are determined by the climatic variables and indicators, including thermal climates, thermal zones, temperature growing periods (LGP_t_), temperature sums (T_sum_), temperature profiles, permafrost evaluation, and potential cropping system.

Step (2). Biomass and yield limited by solar radiation, temperature, and water for all types of crops in the model are calculated for each grid cell. The biomass and yield of all types of crops are calculated under three input and management levels (Table 2) and two water management schemes (rain-fed and irrigated conditions). However, in this research, we only assumed the potential yield was calculated under the highest input and management level. Meanwhile, in the GAEZ model, yield estimation under irrigation conditions assumes the water is sufficient during the crop growth cycle. But under the rain-fed conditions, yield losses will occur due to water stress during the crop growth cycle.

Step (3). Agroclimatic constraints cause losses in the yield and quality of produce. This step revises the results calculated in step (2) by some agroclimatic constraints factors [32]. Table 3 is the list of five different agroclimatic constraints. This step is also based on rain-fed and irrigated conditions.

By combining the five agroclimatic yield reducing factors *fct*_a_, …, *fct*_e_ for constraint types ‘a’ to ‘e’, the reducing factor (*fc*) is calculated:

(1) $$c=\min\left\{\left(1-fct_{a}\right)\times \left(1-fct_{b}\right)\times \left(1-fct_{c}\right)\times \left(1-fct_{d}\right),\;1-fct_{e}\right\}$$

where the *fc* represents the overall yield reducing factor due to agroclimatic constraints ‘a’ to ‘e’, and *fct*_a_*, fct*_b_*, fct*_c_*, fct*_d_*, fct*_e_ are the agroclimatic yield reducing factors for the five constraint types.

Step (4). This step is the calculation of yield reduction due to soil and terrain suitability of each type of crop. This step calculates suitability distributions for each grid-cell by considering all occurring soil-unit and terrain combinations separately. The soil suitability is assessed by seven major soil qualities consisting of nutrient availability, nutrient retention capacity, rooting conditions, oxygen availability to roots, excess salts, toxicity, and workability. These soil qualities are determined by soil characteristics such as soil profile, soil drainage and soil phases. Terrain suitability is estimated from slope and aspect. The calculation is also done separately for rain-fed and irrigated conditions.

Step (5). Computations of step (5) is to read agroclimatic yield calculated for six crop available water capacity (AWC) classes in step (2) and (3), and apply reduction factors due to edaphic evaluation for the specific combinations of soil types and slope classes in step (4).

Step (6). Farmland ratio and irrigation ratio are applied to calculate potential yield of specific crops. In this step, the downscaling method is used to change the 10 km spatial resolution to a more accurate one. In step 5, the yields under rain-fed and irrigation conditions are calculated respectively. In this step, the final yield is calculated according to the following formula within each grid-cell:

(2) $$Y_{t}=Y_{i}\times i+Y_{r}\times \left(1-i\right)$$

where *Y*_t_ represents the total yield of specific crops in each grid-cell (kg/ha), *Y*_i_ and *Y*_r_ represent the potential yield assuming that all the farmland is irrigated land and rain-fed land respectively (kg/ha), and *i* is the irrigation ratio (%) [33].

Therefore, the production can be calculated by:

(3) $$P=Y_{t}\times A_{p}$$

where *P* is the total production (kg), and *A*_p_ represents the crop planting area (ha).

The GAEZ model can also calculate the potential multicropping index, such as 1 (single cropping per year), 2 (double cropping per year), 1.5 (triple cropping for two years), etc. However, winter in Northeast China is long and cold, especially in Heilongjiang Province. Therefore, the potential multicropping index in Northeast China simulated by the GAEZ model is 1.

#### 2.3.2. Spatio-Temporal Dynamics Analysis

Spatio-temporal dynamics analysis of maize potential yield was implemented in Northeast China from 1990 to 2015. First, to analyze the temporal changes of maize potential yield in Northeast China, the results of potential yield and production in Northeast China were simulated by the GAEZ model for three years (1990, 2000, and 2015), and the change curves were produced. Next, to study the spatial dynamics of maize potential yield, we firstly produced the spatial change maps of maize potential yield and maize potential subtypes in two periods (1990–2000 and 2000–2015) at the pixel level, and then analyzed the reasons for spatial changes combining with climate, irrigation ratio, and farmland changes.

#### 2.3.3. Yield Gaps between Actual and Potential Yields

The yield gaps between maize actual and potential yields were calculated. In this study, we calculated the maize potential yield at city scale, and then compared it with maize actual yield from Statistical Yearbook of Northeast China. Then, the rate of actual yield to potential yield was calculated in each city:

(4) $$R=\frac{Y_{a}}{Y_{p}}\times 100\%$$

where *Y*_a_ is the maize actual yield in each city (kg/ha), *Y*_p_ is the maize potential yield in each city, and *R* represents the rate of actual to potential yields in each city (%).

Through the study of yield gaps, we could grasp the maize planting situation in each city, identify the cities with small and large yield gaps, and analyze the reasons.

## 3. Results and Analysis

### 3.1. Validation of the GAEZ Model

**Figure 3:**
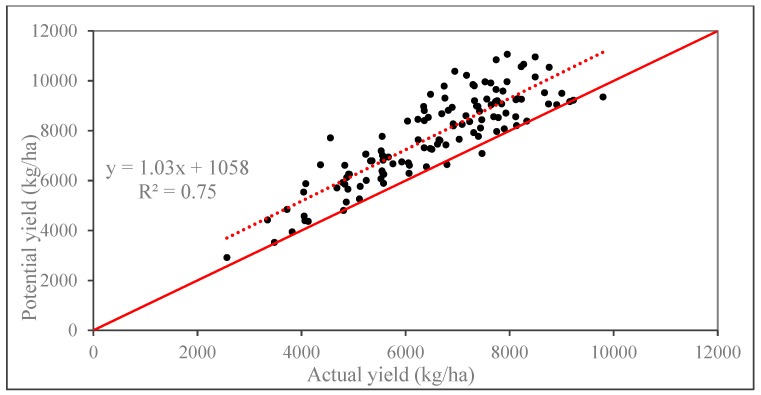
Correlation between maize potential and actual yields of each city in Northeast China from 1990 to 2015.

The simulated potential maize yield of each city in Northeast China for three years was compared with the actual maize statistical yield from Statistical Yearbook to verify the precision of the GAEZ model simulation results, and the regression relation between potential and actual yields was set up (Figure 3). The coefficient of determination (R^2^) was 0.75, indicating that potential and actual yields have a good correlation (Figure 3). Therefore, the trend of simulated potential production was consistent with the trend of actual production. The overall results show an appropriate capability of the GAEZ model to simulate maize potential yield.

### 3.2. Temporal Change of Maize Potential Yield

**Figure 4:**
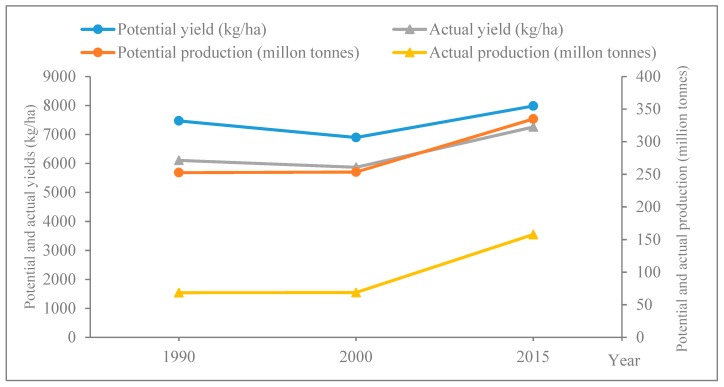
Changes of maize potential and actual yields and production in Northeast China from 1990 to 2015.

The change curves of potential yield and production from 1990 to 2015 are shown in Figure 4 to analyze the temporal changes of maize potential yield in Northeast China. The actual yield and production were also shown as the comparison. It can be seen the maize potential yield and production were all higher than the actual yield and production from 1990 to 2015. Both the potential and actual yields fell slightly from 1990 to 2000 and then rose to 2015. The potential yield decreased from about 7500 kg/ha to 7000 kg/ha in the first decade and then increased gently to around 8000 kg/ha in 2015 (Figure 4). However, both the potential and actual production kept almost unchanged in the first decade and then increased. The potential production was about 260 million tonnes from 1990 to 2000, and then increased sharply to about 340 million tonnes in 2015 (Figure 4).

**Figure 5:**
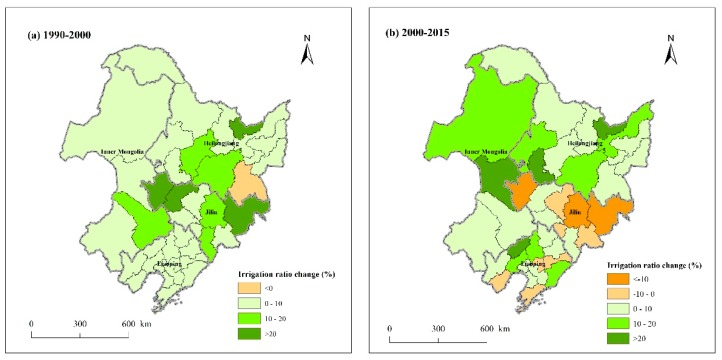
Irrigation ratio changes from 1990 to 2015 in Northeast China. (**a**) 1990–2000; (**b**) 2000–2015.

In this study, the soil and terrain data used to simulate the maize potential yield from 1990 to 2015 were unchanged, so the reasons for the different potential yields and production were mainly the climate change, irrigation ratio change, and farmland area change. Figure 5 showed the irrigation ratio changes during 1990–2000, and 2000–2015 in Northeast China. It can be seen that in the first decade, the irrigation ratio declined in only one city (Figure 5a). In the latter 15 years, the irrigation ratio increased in most cities except for several cities in Liaoning and Jilin (Figure 5b). As for farmland area change, the dryland area was 30.10, 31.97, and 36.00 million tonnes in 1990, 2000, and 2015, respectively, which kept rising continuously. In terms of climate change, we firstly selected ten representative stations in different regions of Northeast China (Harbin, Changchun, Shenyang, and Ulanhot in central region, Wafangdian in south region, Chifeng in southwest region, Right banner of erguna in northwest region, Fujin and Hulin in northeast region, and Mudanjiang in east region), and counted three meteorological factors that mostly affected crop growth (temperature, precipitation, and sunshine duration) of each station for three years. Because the maize growth period in Northeast China is from about April to October, we counted the mean temperature, total precipitation, and total sunshine duration during the seven months, and then calculated the average value at ten stations (Table 4).

From 1990 to 2000, the climate change was evident in Northeast China (Table 4). Both the mean temperature and total precipitation decreased, especially the precipitation that decreased by 140.21 mm. Sunshine duration rose slightly. Although increasing solar radiation and irrigation ratio could help maize absorb more light energy and water in the first decade, they still could not remove the water stress of extreme drop of precipitation, leading to the decrease of biomass during the maize growth cycle and lower actual and potential yields. In the latter 15 years, although the sunshine duration decreased, the large growth of irrigation ratio, temperature, precipitation, and farmland area led to the increase of maize actual and potential yields.

Because production is equal to the yield times planting area (Formula (3)), the reason that the actual and potential production remained almost unchanged in the first decade may be the increase of production caused by the farmland area growth made up for the loss of actual and potential yields caused by climate change. However, because farmland area grew quickly in the latter 15 years, maize actual and potential yields increased gently, and the maize production increased sharply.

### 3.3. Spatial Dynamics of Maize Potential Yield

**Figure 6:**
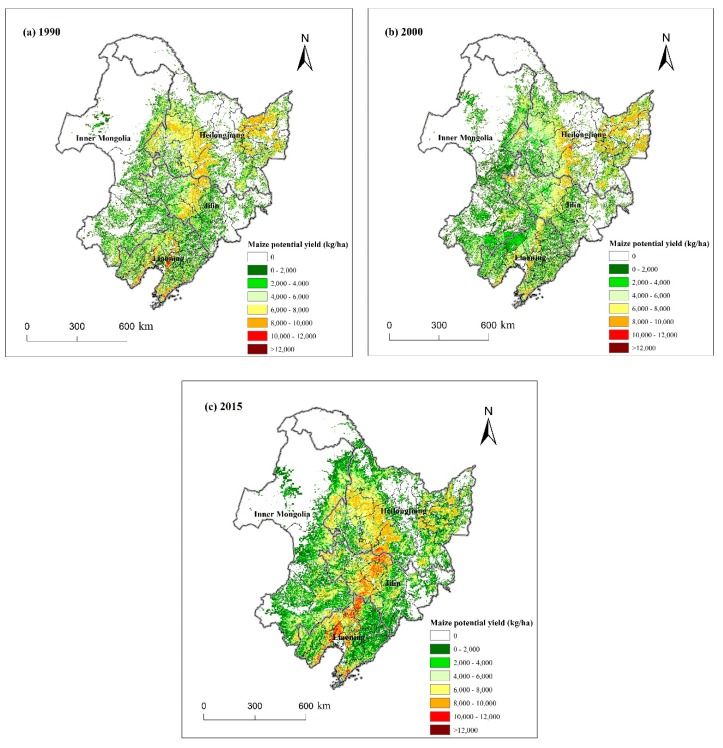
Maize potential yield from 1990 to 2015 in Northeast China. (**a**) 1990; (**b**) 2000; (**c**) 2015.

To study the spatial dynamics of maize potential yield in Northeast China, the spatial distribution maps of maize potential yield for three years were produced (Figure 6a–c). It can be seen that in 1990, 2000, and 2015, the maximum maize potential yields were all over 12,000 kg/ha. Crop potential yield was greatly affected by the terrain. The terrain of the central and northeast regions of Northeast China was relatively flat. We can see the maize potential yield was over 8000 kg/ha for three years that was much higher than that in other regions (such as Inner Mongolia, east regions of Jilin Province, and east and west regions of Liaoning Province, Figure 6). In 2015, it was even more than 10,000 kg/ha in the central region (Figure 6c). In the east and west regions, the potential yields were almost less than 4000 kg/ha for three years in Northeast China (Figure 6).

**Figure 7:**
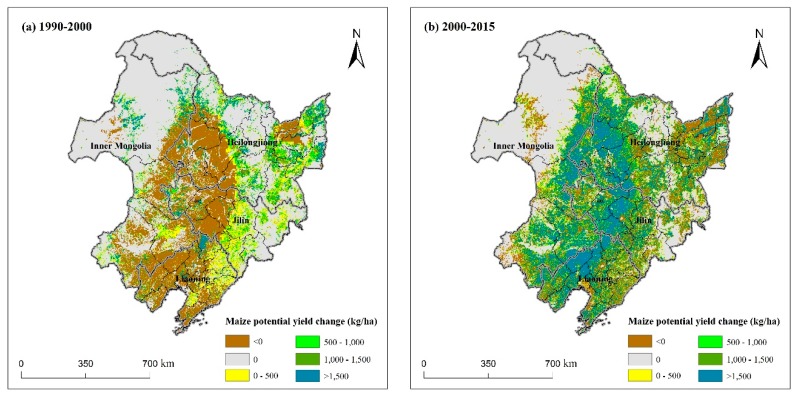
Maize potential yield changes from 1990 to 2015 in Northeast China. (**a**) 1990–2000; (**b**) 2000–2015.

Figure 7 shows the changes of maize potential yield in the two periods at the pixel level. In the first period (from 1990 to 2000), we can clearly see that in most regions of Northeast China, such as the center area (CA), south area (SA), southwest area (SWA), and small regions in northeast area (NEA), the maize potential yield decreased (Figure 7a). However, it increased elsewhere. In the center and east regions of Jilin Province, southeast region of Inner Mongolia, and east region of Liaoning, the maize potential yield increased by less than 500 kg/ha. It increased by 500–1500 kg/ha in some regions of Heilongjiang and even increased by more than 1500 kg/ha in the north of Northeast China.

In the latter 15 years, the potential yield increased by more than 1000 kg/ha in the center area (CA) of Northeast China (Figure 7b), and even more than 1500 kg/ha in some areas. In some regions of northeast area (NEA), east area (EA), and south area (SA) of Northeast China, the maize potential yield decreased.

**Figure 8:**
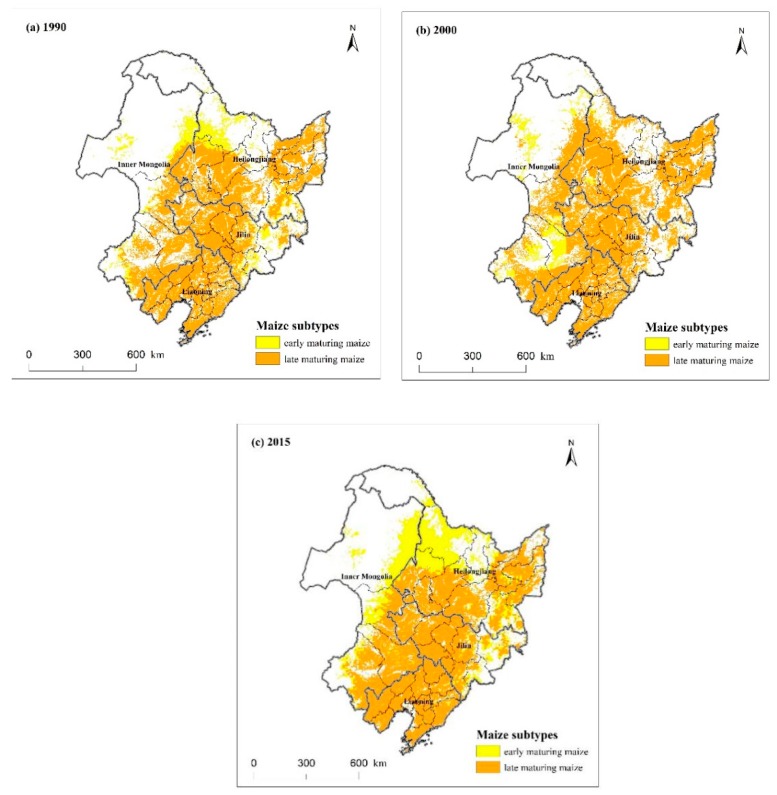
Maize potential subtypes changes from 1990 to 2015. (**a**) 1990; (**b**) 2000; (**c**) 2015.

Maize potential subtypes also changed dramatically from 1990 to 2015 (Figure 8). There were two kinds of maize subtypes, early maturing maize and late maturing maize, simulated by the GAEZ model in years 1990, 2000, and 2015 (Figure 8). The length of growth period (LGP) is closely related to temperature sums of more than 10 °C (Tsum_10_). In Northeast China, early maturing maize grows well in the regions where the Tsum_10_ is 1700–2300 °C, and the LGP is about 110 days. Late maturing maize with the LGP of more than 120 days grows well in the regions where the Tsum_10_ is 2300–3200 °C. We can see in Figure 8 that early maturing maize was distributed in the north area and late maturing maize was distributed in other areas under the influence of Tsum_10_. In 2000, almost all the dryland was suitable for planting late maturing maize except for some regions in the north of Heilongjiang and southeast of Inner Mongolia (Figure 8b). In 1990 and 2015, the area of early maturing maize was larger than that in 2000 (Figure 8a,c).

**Figure 9:**
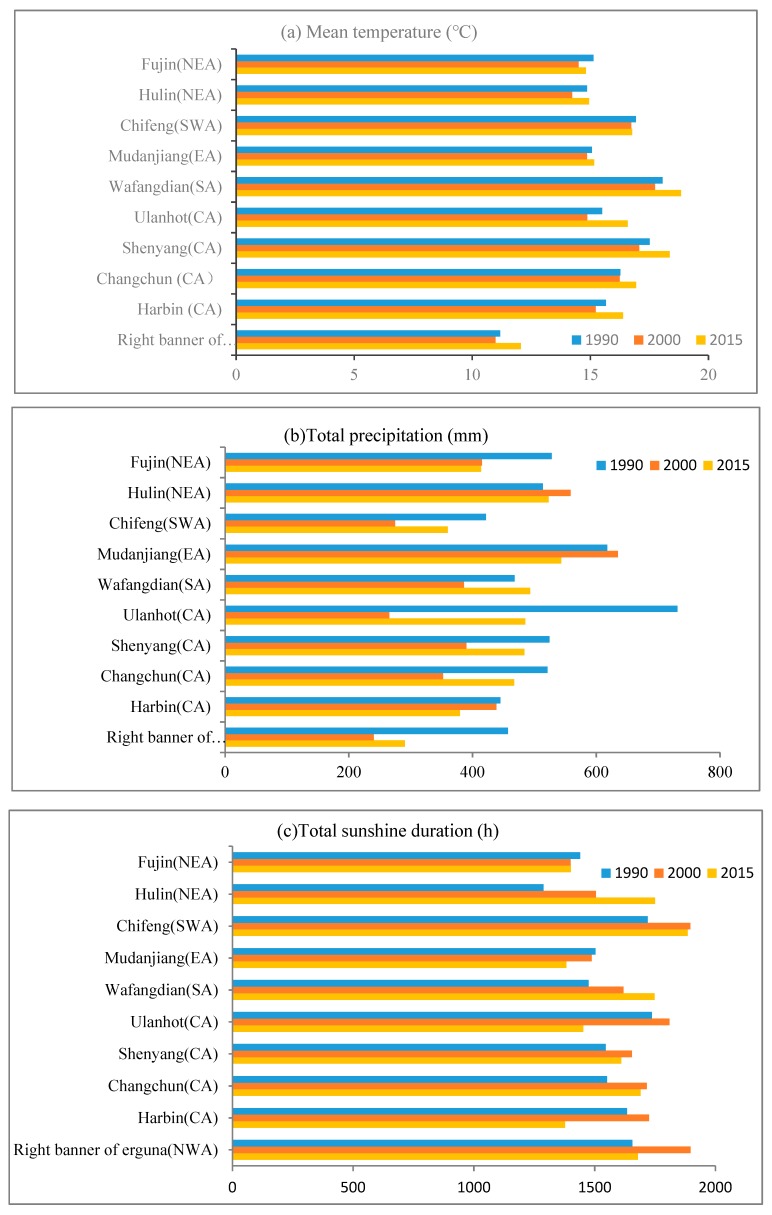
The variation of three meteorological factors for the 10 stations during the maize growth period from 1990 to 2015 in Northeast China. (**a**) Mean temperature; (**b**) Total precipitation; (**c**) Total sunshine duration.

The spatial changes of maize potential yield in the two periods were mainly due to climate change, irrigation ratio change, and farmland change. From 1990 to 2015, irrigation ratio and farmland area basically kept increasing, so climate change was the main reason for maize potential yield changes. We analyzed the variation of three meteorological factors for the ten meteorological stations during the maize growth period (Figure 9). From 1990 to 2000, the mean temperature of all stations decreased in Figure 9a. As for total precipitation, except Hulin in NEA and Mudanjiang in EA, the precipitation of other stations decreased (Figure 9b). The variation of total sunshine duration increased in the first decade except Fujin in NEA and Mudanjiang in EA. Therefore, the reasons that the maize potential yield decreased in the CA, SA, SWA, and small regions in NEA may be cooling damage from lower temperatures and inadequate crop water supply from insufficient rainfall during the maize growth cycle. Although the solar radiation and irrigation ratio increased, it could not make up for the potential yield reduction due to decreasing temperature and rainfall. The maize potential yield increased elsewhere may be due to the increase of precipitation in NEA and EA and the increase of solar radiation in NWA.

From 2000 to 2015, the mean temperature of all stations increased (Figure 9a). Except Fujin and Hulin in NEA, Mudanjiang in EA, and Harbin in CA, the total precipitation of other stations increased (Figure 9b). The total sunshine duration decreased in most stations, except Hulin in NEA and Wafangdian in SA (Figure 9c). In CA, the main reasons that maize potential yield increased by more than 1000 kg/ha were that climate warming promoted maize growth and sufficient precipitation and irrigation ensured the absorption of water. However, the decrease of maize potential yield elsewhere and Harbin in CA was mainly because of inadequate rainfall or solar radiation.

### 3.4. Yield Gaps between Actual and Potential Yields

**Figure 10:**
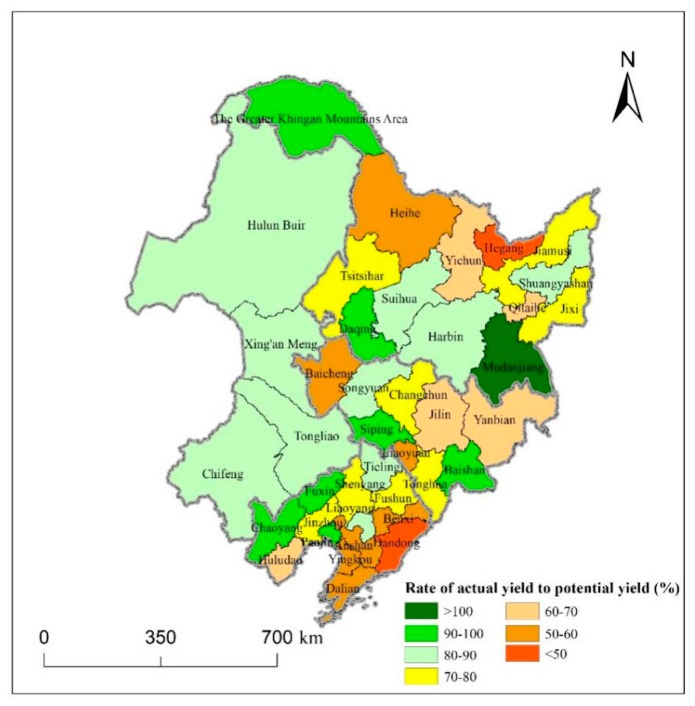
Rate of maize actual yield to potential yield in 2015.

In Part 3.2 and 3.3, we calculated the maize potential yield for three years, studied the spatio-temporal dynamics of maize potential yield from 1990 to 2015 and analyzed the reasons. Also, it is important to explore the yield gaps between maize actual and potential yields. Figure 10 shows the rate of maize actual yield to potential yield in each city in 2015.

It can be seen in Figure 10 that only the actual maize yield in Mudanjiang was higher than the potential yield and the rates of actual yield to potential yield in 17 cities were higher than 80%. This indicates that these cities had high land use efficiency, reasonable selection of maize varieties, and high input and management. These cities were mainly distributed in the east of Inner Mongolia and south of Heilongjiang with flat and low terrain. These regions were more beneficial to the cultivation and growth of field crops such as maize. However, in half of the cities, the actual yields only attained 50−80% of the potential yields in Northeast China. We can see that these cities were mostly located in the plain areas and south region. The reason that the rates were low was that paddy fields in these regions were large, so people paid more attention to the cultivation of rice, ignoring the benefits brought by the planting of maize. Hegang and Dandong were the two cities where the gaps between actual and potential yields were large, and the rates were less than 50%. The two cities need to take urgent measures to improve maize production and shrink the gaps between actual and potential yields.

## 4. Discussions

### 4.1. Limitations of Yield Gaps Analysis

In Section 3.4, we analyzed the yield gaps between maize actual and potential yields in each city by calculating the rate of actual yield to potential yield, and identified the cities where the yield needs to be boosted. However, the yield gap analysis still had a few limitations. Firstly, the GAEZ model can calculate the crop potential yield and potential multicropping index under existing topography, soil, and climate conditions. In fact, farmers usually decide the cropping system according to their previous planting experience. Although the maize potential multicropping index calculated by the GAEZ model in Northeast China was 1 (single cropping per year), which was consistent with the actual situation, the potential multicropping index calculated by the GAEZ model in other regions may be 1.5 or 2, which may be different from the actual cropping system. Therefore, the actual yield will be different from the potential yield, thus leading to inaccurate results. Secondly, since the GAEZ model can calculate the subtypes and distribution of specific crop to obtain the potential yield, the results of crop subtypes may be different from the actual crop subtypes planted by farmers, which could result in different LGP. Comparing potential and actual crop yields based on the same LGP can make the results more accurate. This is one of the limitations of the yield gap analysis which needs to be addressed in the future.

### 4.2. Several Other Crop Production Models

Modelling crop growth is a tradition starting long before today’s computer models [34]. So far, different models have been explored by many scholars to study crop production. Some models employ detailed representations of plant phenology and physiology, resulting in parameterization and calibration. For instance, the Environmental Policy Integrated Climate (EPIC) model was developed to simulate plant and soil ecological systems including the processes of weather, crop growth, crop and soil management, tillage, soil temperature, carbon cycling, nutrient cycling (N, P, K), soil erosion, hydrology, and soil water dynamics [35,36,37,38]. WOFOST is another method for analyzing the growth and production of crops under a wide range of weather (such as temperature and radiation) and soil conditions (for example, soil moisture and soil types) [39,40].

Another group of models pays more attention to soil biogeochemistry and nutrient cycling, such as the Denitrification and Decomposition (DNDC) model [41] and Carnegie–Ames–Stanford Approach (CASA) model [42]. The DNDC model is a process-oriented simulation tool of soil carbon and nitrogen based on biogeochemistry that has been discussed in detail elsewhere [43]. The CASA model is an excellent process-based model to estimate the land plant NPP based on the plant growing mechanism [44].

Although the calculation methods are different, many important factors have been considered in the above models, such as climate, soil moisture and attributes, and crop management. However, besides light, temperature, water, and soil, terrain is also crucial to obtain crop potential yield and production. Relatively speaking, flat farmland is more suitable for most crop growth. Farmland with a large slope has relatively low crop yield due to serious soil nutrient and moisture loss. Therefore, the above models didn’t take into account the effect of terrain on crop yield, which is the disadvantage of these models.

### 4.3. The Advantages and Limitations of the GAEZ Model

Compared with other models, we suppose the GAEZ model can simulate more accurate results regarding crop potential yield. The GAEZ model comprehensively considers the radiation, temperature, and other climatic factors that affect the crop growth, such as the length of the growing season, the water needs in different growth stages, etc. [45]. More importantly, the GAEZ model considers the impact of soil and terrain on crop growth.

The GAEZ model also has some limitations. First, in this model, we usually assume the potential production under the highest input and management level and define corresponding Leaf Area Index (LAI) and Harvest Index (HI) of crops, and then obtain the maximum production (Table 2). However, it’s very difficult to realize the highest input and management in all regions during crop growth. To be more accurate, the model should modify the parameters of input and management levels according to the characteristics of different regions. Second, in this study, the meteorological data for 1990–2015 were obtained from 99 national meteorological stations maintained by the Chinese Meteorological Administration and were interpolated to produce a continuous surface. However, to obtain a very high-resolution and accurate spatial distribution map by spatial interpolation was very difficult because the meteorological stations were scarce within the region [46]. Third, monthly mean climatic data were integrated into the GAEZ model, but extreme climate conditions, such as extreme temperature and precipitation, may have had large effects on crop potential yield [29]. These extreme climate conditions have not been considered in this model.

## 5. Conclusions

In recent decades, as the major crop, maize consumption has increased sharply in Northeast China. By using the GAEZ model to investigate the spatio-temporal dynamics of maize potential yield and yield gaps between actual and potential yields in Northeast China from 1990 and 2015, we found that the maize potential yield decreased from about 7500 kg/ha to 7000 kg/ha in the first decade, mainly due to the lack of water in the growth cycle caused by the decrease of precipitation. The potential production remained unchanged, which was because the growth of high-quality farmland made up for the loss of yield caused by climate change. In the latter 15 years, the large growth of temperature, irrigation ratio, precipitation, and farmland area led to the increase of maize potential yield and production.

The spatial variation of maize potential yield was also significant from 1990 to 2015, and climate factors, especially temperature, precipitation, and solar radiation, had great effect on maize potential yield. In the first period, the maize potential yield decreased in most regions of Northeast China, such as CA, SA, SWA, and small regions in NEA, due to cooling damage from lower temperature and inadequate crop water supply from insufficient rainfall during maize growth cycle in these regions. The reason that the maize potential yield increased elsewhere may be the increase of precipitation in NEA and EA and the increase of solar radiation in NWA. In the second period, the main reasons that the maize potential yield increased by more than 1000 kg/ha in CA may be that climate warming promoted maize growth and sufficient precipitation and irrigation ensured the absorption of water. However, the decrease of maize potential yield elsewhere and Harbin in CA was mainly because of inadequate rainfall or solar radiation.

In terms of the yield gaps between maize actual and potential yields, only the actual maize yield in Mudanjiang was higher than the potential yield. The rates of actual yield to potential yield in 17 cities were higher than 80%. In half of the cities that are mostly located in the plain areas and south region, the actual yields only attained 50–80% of the potential yields in Northeast China. The reason may be that people paid more attention to the cultivation of rice, ignoring the benefits brought by the planting of maize in these regions. Hegang and Dandong need to take urgent measures to improve maize production and shrink the yield gaps between actual and potential yields, because the gaps between actual and potential yields were large.

Overall, the spatio-temporal changes of maize potential yield in Northeast China were great from 1990 to 2015, but more attention should be paid to the yield gaps between actual and potential yields. Some regions still need to take urgent measures to improve maize yield. Although increasing farmland area can help to increase crop production, only increasing the amount of farmland would damage the ecological environment. The key to ensuring food security is to improve crop yield. To do this, people should ensure more investment and management to close the gaps, such as establishing basic farmland protection areas, increasing irrigation area to supply more water, adopting mechanized farming, and increasing weeding and fertilization as much as possible. Meanwhile, it is also essential to cope with climate change actively, optimize crop structure reasonably and improve the farmland utilization efficiency urgently.

## Figures and Tables

**Table 1:** Actual maize production and total grain production in Northeast China in 2015.

Province	Total Grain Production (Million Tonnes)	Actual Maize Production (Million Tonnes)	Rate of Maize Production to Total Grain Production (%)
Heilongjiang	63.24	35.44	56.04
Jilin	36.47	28.06	76.94
Liaoning	20.02	14.04	70.13
Inner Mongolia	22.62	18.19	80.01
Northeast China	142.35	95.73	67.25

**Table 2:** Input level for the Global Agro-ecological Zones (GAEZ) model.

Input level	Explanations
Low	Traditional cultivars, labor intensive techniques, and no application of nutrients and chemicals for pest and disease control
Medium	Medium labor intensive, some fertilizer application and chemical pest disease and weed control.
High	Low labor intensity and application of nutrients and chemical pest disease and weed control.

**Table 3:** Five agroclimatic constrains.

Agroclimatic constraints	Explanations
a	Long-term limitation to crop performance due to year-to-year rainfall variability
b	Pests, diseases, and weeds damage on plant growth
c	Pests, diseases, and weeds damage on quality of product
d	Climatic factors affecting the efficiency of farming operations
e	Frost hazards

**Table 4:** Average value of three meteorological factors at ten stations during the maize growth period from 1990 to 2015.

Year	Mean Temperature (°C)	Total Precipitation (mm)	Total Sunshine Duration (h)
1990	16.61	522.95	1555.11
2000	16.17	382.74	1631.36
2015	17.08	452.19	1611.93
